# Impact of putatively beneficial genomic loci on gene expression in little brown bats (*Myotis lucifugus*, Le Conte, 1831) affected by white‐nose syndrome

**DOI:** 10.1111/eva.13748

**Published:** 2024-09-19

**Authors:** Robert Kwait, Malin L. Pinsky, Sarah Gignoux‐Wolfsohn, Evan A. Eskew, Kathleen Kerwin, Brooke Maslo

**Affiliations:** ^1^ Department of Ecology, Evolution and Natural Resources Rutgers, The State University of New Jersey New Brunswick New Jersey USA; ^2^ Department of Ecology and Evolutionary Biology University of California Santa Cruz Santa Cruz California USA; ^3^ Department of Biological Sciences University of Massachusetts Lowell Massachusetts USA; ^4^ Institute for Interdisciplinary Data Sciences University of Idaho Moscow Idaho USA

**Keywords:** disease, eQTL, evolution, expression, *Myotis lucifugus*, White‐nose syndrome

## Abstract

Genome‐wide scans for selection have become a popular tool for investigating evolutionary responses in wildlife to emerging diseases. However, genome scans are susceptible to false positives and do little to demonstrate specific mechanisms by which loci impact survival. Linking putatively resistant genotypes to observable phenotypes increases confidence in genome scan results and provides evidence of survival mechanisms that can guide conservation and management efforts. Here we used an expression quantitative trait loci (eQTL) analysis to uncover relationships between gene expression and alleles associated with the survival of little brown bats (*Myotis lucifugus*) despite infection with the causative agent of white‐nose syndrome. We found that 25 of the 63 single‐nucleotide polymorphisms (SNPs) associated with survival were related to gene expression in wing tissue. The differentially expressed genes have functional annotations associated with the innate immune system, metabolism, circadian rhythms, and the cellular response to stress. In addition, we observed differential expression of multiple genes with survival implications related to loci in linkage disequilibrium with focal SNPs. Together, these findings support the selective function of these loci and suggest that part of the mechanism driving survival may be the alteration of immune and other responses in epithelial tissue.

## 1 INTRODUCTION

Anthropogenic environmental change disrupts ecological communities globally and threatens the viability of populations (Hannah, 2021). The resulting stressors, such as disease, biological invasions, and climate change, can cause demographic declines that require population‐level adaptation to avoid extinction. Rapid evolution is often documented by identifying alleles that have changed in frequency following a mass mortality event. For example, green anole lizards (*Anolis carolinensis*, Voigt, 1832) surviving a severe winter storm in the southern United States exhibited genomic and transcriptional changes (Campbell‐Staton et al., 2017), European rabbit (*Oryctolagus cuniculus*, Linnaeus, 1758) populations in Australia, France, and the United Kingdom underwent rapid selection after the introduction of the myxoma virus (Alves et al., 2019), and Tasmanian devils (*Sarcophilus harrisii*, Harris, 1808) experienced significant allele frequency changes after the emergence of facial tumor disease (Epstein et al., 2016).

Identification of beneficial loci can be instrumental for the conservation of imperiled species. For example, determining whether genetic adaptation is responsible for differential survival is useful because host response to disturbance impacts the likelihood of successful management intervention (Maslo et al., 2017). Further, in the case of wildlife disease, frequencies of resistant alleles can be used to assess the susceptibility of naive populations, and individuals with resistant genotypes can be translocated to vulnerable populations (Aitken & Whitlock, 2013; Whiteley et al., 2015). Such genetic rescue initiatives remain underused and have rarely targeted particular beneficial alleles, likely due to the recency of widely available molecular technology, a lack of certainty in the adaptive potential of loci, and a lag in translating research into management action (Cullingham et al., 2023; Fitzpatrick et al., 2023; Funk et al., 2012; Whiteley et al., 2015). However, translocations from healthy populations have effectively mitigated demographic stress caused by low genetic diversity, in some cases with remarkably few individuals (*Puma concolor*, Linaeus, 1771; Johnson et al., 2010; *Neotoma magister*, Baird, 1857; Smyser et al., 2013). Therefore, where inbreeding depression is the primary stressor, nonspecific genetic rescue can be effective. However, in cases where populations are experiencing abrupt environmental change, increasing genetic diversity without consideration of allele‐specific impacts on survival may be ineffective.

Genome‐wide scans for selection (GWSS) are used to identify adaptive loci within populations (Gondro et al., 2013). Selective sweeps can be identified by searching for genomic regions or loci exhibiting allele frequency changes greater than would be expected by drift or gene flow alone across time points before and after a perturbation. Despite their utility, GWSS can produce false positive signals (De Mita et al., 2013; François et al., 2016). The large number of tests required to compare the effect of alleles at each locus can produce multiple testing errors (De Mita et al., 2013; François et al., 2016; Nunes et al., 2011). In addition, analyses can suffer from error due to linkage disequilibrium (LD), whereby loci in close proximity do not assort independently during recombination, resulting in linked inheritance of alleles (Gaut & Long, 2003; Li et al., 2021; Nordborg & Tavaré, 2002). If LD is not accounted for, GWSS produce a scale of investigation at the level of linkage group rather than loci, which is insufficient for management efforts targeting specific genotypes. Finally, GWSS do little to demonstrate mechanisms by which loci impact phenotypes. Genotype‐to‐phenotype connections are a current frontier in genomics and can aid in conservation efforts, particularly because the phenotypic trait is what actually gets acted upon by selection and promotes survival or mortality (Bomblies & Peichel, 2022; Dalziel et al., 2009; Matthews et al., 2023). In addition, phenotypic variation is regulated by environmental parameters, which may act in concert with or oppose variation associated with genome sequence (Matthews et al., 2023; Stapley et al., 2010). A particular genotype may be suitable in one environment yet lead to decreased fitness in another. Without understanding the mechanism by which genomic loci impact phenotypic traits, it is difficult to predict the success of genotype transplants to susceptible populations or the impact of the same disturbance on other species.

To evaluate the results of GWSS more fully, functional validation or experimental demonstration is often required (Bomblies & Peichel, 2022; Cullingham et al., 2020; Matthews et al., 2023; Salvi & Tuberosa, 2005; Voelckel et al., 2017). In model systems, lab‐based genetic manipulation studies are feasible. However, this methodology is often infeasible for imperiled wild populations.

One potential approach for reducing the uncertainty associated with GWSS is complementary validation, a term coined in Cullingham et al. (2020), in which a stronger evidence base for a hypothesized host adaptation is generated by identifying relationships between loci putatively under selection and their phenotypic function at multiple physiological scales (Cullingham et al., 2020). Further, investigating loci in genomic regions surrounding loci putatively under selection can help reduce the scale of inference from linkage groups to loci under selection by building evidence over multiple studies. A greater correlation between one locus within a linkage group and phenotypic function at multiple scales can offer convincing evidence supporting the beneficial function of that locus. Finally, complementary validation simultaneously provides evidence of the mechanisms by which loci under selection promote persistence by using genotype‐to‐phenotype connections for validation (Cullingham et al., 2020; Matthews et al., 2023; Voelckel et al., 2017). In short, connecting genotype to phenotype is useful both for validating loci putatively under selection and for beginning to determine their mechanism of action.

Little brown bats (*Myotis lucifugus*, Le Conte, 1831) affected by white‐nose syndrome (WNS) are an appropriate case study for a complementary validation approach. WNS is a devastating fungal disease that has killed millions of bats in North America since its discovery in 2006 (Cheng et al., 2021; Frick et al., 2010). The pathogenic agent of WNS, *Pseudogymnoascus destructans* (Minnis, 2013), invades the exposed epidermal tissue of bats during hibernation (Lorch et al., 2011; Warnecke et al., 2012). The damage caused by fungal growth does not directly lead to mortality; rather, infection initiates a cascade of physiological disruptions that act to increase the frequency of arousal from torpor during hibernation (Reeder et al., 2012; Verant et al., 2014; Warnecke et al., 2013). Arousals from torpor are part of the natural hibernation cycle but are energetically expensive and taxing on fat and water stores (Thomas et al., 1990; Thomas & Cloutier, 1992). Elevated torpor arousal frequencies can quickly deplete energy reserves and lead to death (Cheng et al., 2019; Reeder et al., 2012; Willis et al., 2011).

Despite experiencing catastrophic initial declines from WNS, *M. lucifugus* populations have stabilized near the epicenter of disease outbreak (Hoyt et al., 2021; Maslo et al., 2015; Reichard et al., 2014). Studies of persisting populations demonstrate that WNS survivors exhibit two phenotypic phenomena. First, the mean torpor bout duration of infected but persisting individuals is no different from that of healthy, unexposed bats (Frank et al., 2019; Lilley et al., 2016). Second, comparisons of hibernating colonies before and after WNS emergence show that body fat composition is greater for bats persisting with disease (Cheng et al., 2019). In addition to research on physiological traits, multiple GWSS have identified over 100 single‐nucleotide polymorphisms (SNPs) putatively under selection in *M. lucifugus* due to WNS (Auteri & Knowles, 2020; Donaldson et al., 2017; Gignoux‐Wolfsohn et al., 2021; Lilley et al., 2020). Intriguingly, many of the SNPs appear within genes or genomic regions related to the regulation of torpor, metabolism, body composition, and immune function (Auteri & Knowles, 2020; Donaldson et al., 2017; Gignoux‐Wolfsohn et al., 2021; Lilley et al., 2020). However, no study has attempted to independently validate the proposed adaptive genetic variation or directly associate it with phenotypes that promote persistence.

Like many functionally important loci, most loci putatively related to WNS survival are not in coding regions and thus do not influence phenotype by altering protein structure (Barrett et al., 2012; Brown et al., 2013; De Gobbi et al., 2006; Zaugg et al., 2022). Rather, such loci can act as transcriptional regulators that alter gene expression (Brown et al., 2013; De Gobbi et al., 2006; Zaugg et al., 2022). One way of demonstrating the relationship between loci and transcription is an expression quantitative trait loci (eQTL) analysis (Brown et al., 2013; Shi, 2020). An eQTL analysis compares transcript counts for each gene to allele identities at a group of loci to determine if allelic differences at any loci correlate with expression levels of other genes (Shi, 2020). Functional annotation of differentially expressed genes can then be used to infer the phenotypic consequences of the loci.

Here, we employed an eQTL analysis to validate previously identified SNPs putatively under selection in little brown bats and uncover clues regarding their mechanism of action that may be relevant to WNS. We also performed an eQTL analysis on additional SNPs near previously identified loci to determine if these flanking SNPs possess potentially beneficial functions that may have been masked by LD.

## 2 METHODS

### 2.1 White‐nose syndrome (WNS) pathology

WNS is an organism‐level pathology involving interactions between multiple tissues. Several mechanisms linking pathogen propagation to increased torpor arousal frequency have been proposed, including hypercapnia, increased evaporative water loss across damaged tissue, and electrolyte imbalance (Cryan et al., 2010; Verant et al., 2014; Warnecke et al., 2013; Willis et al., 2011).

Transcriptomic studies comparing the wing tissue of exposed and unexposed bats suggest that in *M. lucifugus*, *P. destructans* initiates a typical mammalian response to fungal infection (Davy et al., 2017, 2020; Field et al., 2015, 2018; Lilley et al., 2019; Romani, 2011; Whiting‐Fawcett et al., 2021). First, *P. destructans* is recognized by C‐type lectin and toll‐like receptors in the epithelium, which activate transcription factors like NF‐kβ within keratinocytes of the skin (Davy et al., 2020; Field et al., 2015, 2018; Whiting‐Fawcett et al., 2021). These factors induce the transcription of cytokines and chemokines (immune signaling molecules) that activate local inflammatory and immune responses (Davy et al., 2020; Field et al., 2015, 2018). A systemic response including inflammation, cytokine signaling, and leukocyte activity follows local immune signaling (Davy et al., 2020; Field et al., 2018; Lilley et al., 2019; Rapin et al., 2014; Whiting‐Fawcett et al., 2021). Unlike normothermic mammals, hibernating bats do not appear to invoke a full adaptive immune response upon infection, likely due to suppression of those pathways during torpor (Field et al., 2015, 2018; Whiting‐Fawcett et al., 2021). Upon arousal *M. lucifugus* reactivates its' adaptive immune system by commencing interleukin, antibody, and neutrophil responses, but these pathways appear ineffective at combating *P. destructans* infection (Field et al., 2018; Lilley et al., 2017; Whiting‐Fawcett et al., 2021). Additionally, upon arousal, some bats appear to experience a systemic inflammatory response, where the sudden resurgence in immune activity after hibernation‐related immunosuppression can lead to mortality (Meteyer et al., 2012). Outside of the immune system, infection causes changes related to lipid and carbohydrate cycling and metabolism in *M. lucifugus* (Field et al., 2015, 2018). Metabolic responses to infection have been noted in other wildlife disease systems involving fungal dermatitis (Eskew et al., 2018).

### 2.2 Sample collection and preparation

We conducted our eQTL analysis on wing tissue. We chose the wing membrane because multiple transcriptomic studies have already described molecular responses to *P. destructans* infection in this tissue (Davy et al., 2017, 2020; Field et al., 2015, 2018; Lilley et al., 2019). In addition, the epidermis of the wing is the primary point of contact between host and pathogen in the WNS system, so systemic responses likely originate within this tissue (Meteyer et al., 2009; Verant et al., 2014; Warnecke et al., 2013). Finally, wing biopsies can be obtained without sacrificing the animal, which is an important consideration when studying a species of conservation concern. We focused on a subset of available putatively beneficial SNPs, namely those uncovered in Gignoux‐Wolfsohn et al. (2021), because (1) a greater number of SNPs requires more tests and therefore lowers statistical power; (2) these loci were generated from a whole‐genome analysis; and (3) they are located near and within genes that have implications for WNS pathophysiology.

**FIGURE 1:**
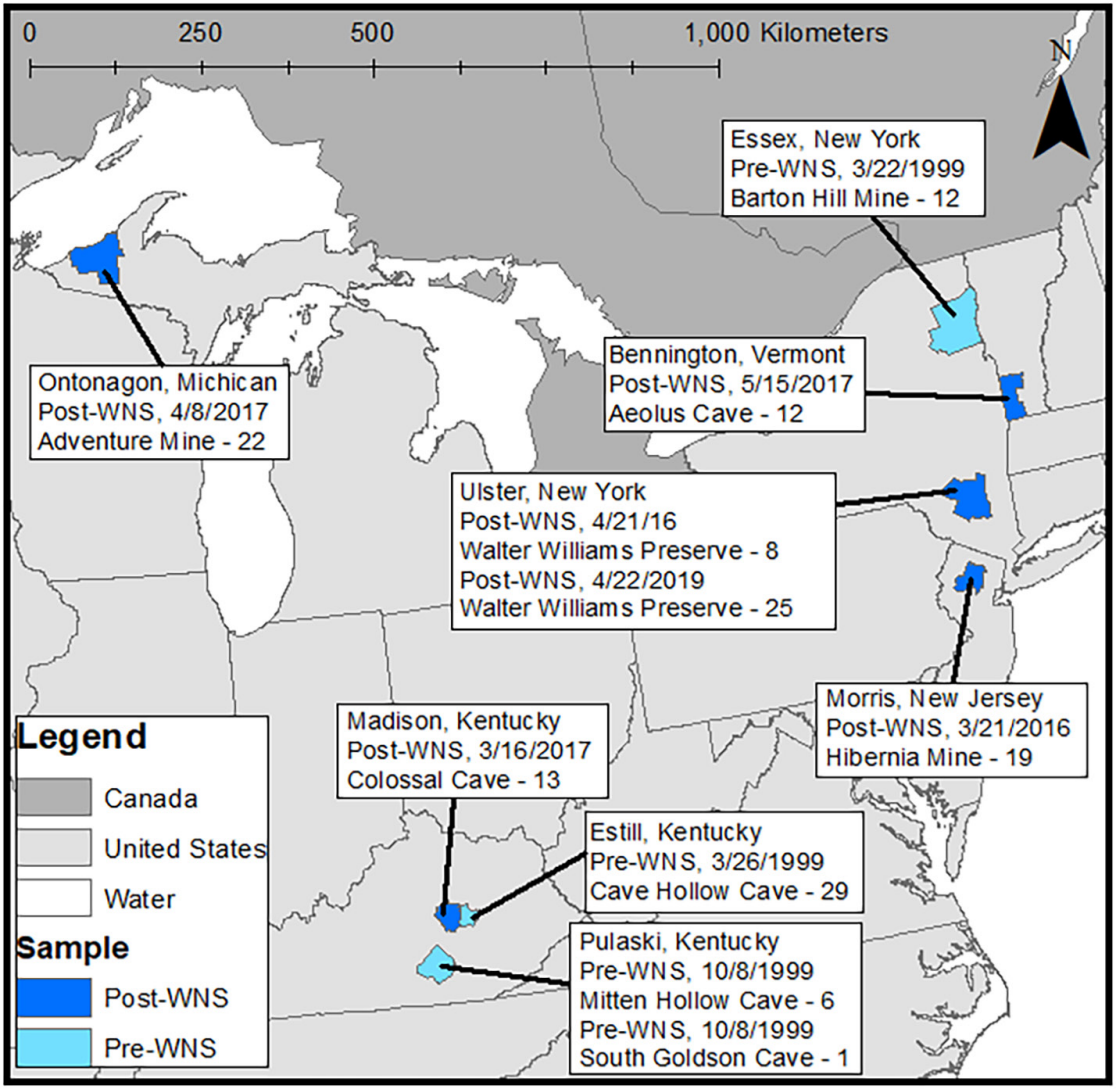
A map of the eastern United States showing counties from which samples were collected. All samples were collected from torpid bats on hibernacula walls.

We obtained wing tissue samples of *M. lucifugus* collected in 1999 prior to WNS emergence from four hibernation sites across the U.S., including Estill and Pulaski counties in Kentucky, and Essex County, New York (Figure 1). These samples were collected from torpid bats during the hibernation season, immediately preserved in dimethyl sulfoxide (DMSO), and stored in a −80°C freezer at Western Michigan University (M. Vonhof, personal communication). We collected the remaining samples from WNS‐infected sites that support colonies of hibernating *M. lucifugus*, located in Kentucky (Madison County), Michigan (Ontonagon County), New Jersey (Morris County), New York (Ulster County), and Vermont (Bennington County). We collected post‐WNS samples (defined as sites with confirmed infection for at least 2 years) from torpid bats during the hibernation seasons of 2016 and 2017 by gently removing individuals from the hibernaculum substrate, extending a wing, and using a sterile biopsy punch to collect a 3‐mm sample from the wing. We immediately placed tissue samples into 1.5‐mL microcentrifuge tubes filled with RNAlater (Ambion Inc., Austin, TX), transported them to Rutgers University, and stored them at −80°C. All post‐WNS samples were collected in close coordination with relevant state wildlife agencies under the appropriate permits and approvals (Rutgers IACUC Protocol #: 999900205). The full dataset (Table S1) is representative of both the *P. destructans*‐infected range of *M. lucifugus* as well as a gradient of WNS infection histories. In total, we gathered 147 samples consisting of 48 pre‐WNS and 99 post‐WNS individuals.

Prior to extraction, we cut each tissue sample in half using tweezers and a scalpel on a hard plastic cutting board. To avoid cross‐contamination, we flame‐sterilized instruments and sterilized the cutting board with 10% bleach between each sample.

### 2.3 Genotyping bats at target single‐nucleotide polymorphisms (SNPs)

We extracted DNA from one half of the sample using the QIAamp DNA micro kit (Qiagen, Hilden, Germany) following the manufacturer's protocols. To increase DNA yield, we also included a 10‐min elution buffer incubation step, followed by a 10‐min elution centrifugation step. We constructed sequencing libraries using the Nextera DNA flex kit (Illumina, San Diego, CA) following the manufacturer's protocols. Following DNA library construction, we enriched the libraries for 2000‐bp regions (*hereafter*, target regions) centered on target SNPs using tiled 80‐bp molecular probes designed as part of a custom myBaits kit (Arbor Scientific, Ann Arbor, MI). We designed the kit around the 63 SNPs (Table S2) identified by Gignoux‐Wolfsohn et al. (2021). To compare gene expression patterns in target SNPs and regions relative to those occurring across the entire genome, we included in our probe set 1000 regions of 1000 bp each centered on random sites (*hereafter*, random regions) throughout the genome. Prior to beginning the probe protocol, we pooled samples in equimolar ratios into groups of four to five and performed the biotin clean‐up step detailed in appendix A2 of the myBaits protocol. Because libraries prepared using Nextera Flex kits tend to show increased streptavidin affinity, which can impact capture efficiency, the cleanup protocol is intended to treat libraries such that they are compatible with the myBaits protocol. Following enrichment, we pooled the enriched libraries in equimolar ratios into two batches and sequenced them on two lanes of an Illumina NovaSeq SP Lane v1.5 with a read length of 300 nt.

Raw reads passing the sequencing quality filter were demultiplexed using BARCODESPLITTER and processed on the Amarel computer cluster at Rutgers University (Leach & Parsons, 2019). We removed optical duplicates using *clumpify* from the *bbtools* package with the *dedupe* optical option activated and with *dedist* specified at 1200 as is recommended for NovaSeq libraries (Bushnell, 2014). We then used TRIMMOMATIC to remove adapter sequences, reads with average Phred scores lower than 33, reads with a 4 bp sliding window Phred score average of lower than 15, and sequences with trimmed lengths lower than 30 (Bolger et al., 2014). Contaminant reads aligning to human, mouse, bacteria, fungi, and viruses from GenBank were also eliminated using *fastq*‐screen (Wingett & Andrews, 2018). We removed pair mates for which the other read in the pair was filtered as a contaminant using *repair*.sh from the *bbtools* package (Bushnell, 2014). The remaining reads were aligned to the reference *M. lucifugus* genome (myoluc2.0 in GenBank) using *hisat2* (Kim et al., 2019). Prior to alignment, we masked regions of the myoluc2.0 genome that consisted of repeats or returned mappability scores lower than 1 using GenMap and a *repeatmasker* (Nishimura, 2000; Pockrandt et al., 2020). After alignment, we converted the resulting SAM files to BAM files, then sorted and indexed them using SAMtools (Li et al., 2009).

### 2.4 Quantification of gene expression

We extracted RNA from the second half of each wing biopsy sample using the RNeasy Micro kit (Qiagen, Hilden, Germany) following the manufacturer's instructions. We performed DNase digestion in a tube. Prior to beginning each extraction, we sterilized the lab bench and supplies with a 10% bleach solution. For library prep, we used the Quant‐seq 3′ mRNA‐seq FWD kit (Lexogen, Wien, Austria). This method is equivalent to total mRNA‐seq for expression count‐based analyses and allows for a reduced sequencing cost by only targeting the 3′ end of mRNA transcripts. The Quant‐seq kit starts with the reverse‐transcription of RNA into cDNA and rRNA removal. We then added adapters and sample‐specific indexes to one end of the RNA transcripts via a PCR that targets poly‐A tails. We performed a bead‐based cleanup and size selection, pooled samples in equimolar ratios into two batches, and performed single‐end sequencing on two lanes of an Illumina NovaSeq SP lane v1.5 with a read length of 100 nt.

Raw reads that passed the quality filter were demultiplexed using BARCODESPLITTER and downloaded to the Amarel computer cluster at Rutgers University (Leach & Parsons, 2019). Because pre‐ and post‐WNS samples were preserved in different media, we first conducted several bioinformatic tests of quality, including comparing the number of uncalled bases and raw read counts between sample groups. The results of these analyses suggest that all samples were of comparable quality (Table S3). We also elected to include seven pre‐WNS samples that were collected during hibernation in the late fall rather than early spring to maximize our sample size (Table S1). To account for potential transcriptomic differences between sampling periods, we included season as a covariate in all analyses.

For all samples, we removed residual rRNA contamination with the function *bbduk* from the *bbtools* package (Bushnell, 2014) using the SILVA reference rRNA libraries SSU and LSU, which contain curated rRNA sequences from multiple taxa, including bacteria and humans (Quast et al., 2012). We removed beginning and end bases with quality scores lower than 3, reads with sections of group quality scores lower than 15 (sliding window 4:15), poly‐A tails, adapters, and reads of fewer than 36 bp using TRIMMOMATIC (Bolger et al., 2014). We then removed reads that aligned to sequences of humans, mice, bacteria, fungi, and viruses in GenBank using FastQscreen (Wingett & Andrews, 2018) and checked reads for quality using *fastqc* (Andrews, 2010). Filtered reads were aligned to the reference *M. lucifugus* genome (myoluc2.0) using the STAR aligner (Dobin et al., 2013). We generated count tables from read alignments using HT‐Seq (Anders et al., 2015) and concatenated the read tables into one file. We removed transcripts with counts of fewer than 147 (the number of samples in the study) across all samples and those that did not have at least 10 counts in at least one sample in R using the package *dplyr* (Team, 2013; Wickham et al., 2015). We normalized (trimmed mean of M‐values; TMM) reads using the R package *edgeR* (Robinson et al., 2010). The TMM method of normalization has been found to be ideal for eQTL analyses because it is robust to variability in library size and constitution (Robinson & Oshlack, 2010; Yang et al., 2021). Finally, we performed a PCA in R to visually identify outliers (Vu, 2011).

### 2.5 Isolated target SNP expression quantitative trait loci (eQTL) analysis

#### 2.5.1 Variant calling

We performed an eQTL analysis on the target SNPs in isolation to test for correlations with gene expression in wing tissue. We called SNPs at the loci of interest using *angsd* with the sites option to genotype only at target SNPs (Korneliussen et al., 2014), setting a genotype likelihood threshold of >80%. We excluded sites if they had less than 20× coverage, minor allele frequency (MAF) <5%, fewer than five heterozygotes, or were missing in more than 5% of samples. We subsequently removed samples if they consisted of more than half missed calls, and we removed SNPs if they were missing in more than half of the samples.

#### 2.5.2 eQTL analysis

We used the R package MatrixEQTL to run a cis/trans eQTL analysis with an ANOVA model (Shabalin, 2012), considering any genes within 1 × 10^6^ base pairs of the target SNP to be in cis, the default setting for MatrixEQTL. We set the p‐value cutoffs at 2 × 10^−2^ and 1 × 10^−2^ for cis and trans eQTLs, respectively, and included sex, hibernaculum, season, period (before or after WNS emergence), and RNA sequencing batch as covariates. We included hibernaculum as a covariate to control for potential differences in gene expression related to population structure and season to account for potential differences related to six samples collected during early hibernation rather than late hibernation.

We also ran an iteration of this eQTL only including the post‐WNS samples. Because we are uncertain of the impact these target loci have on bat phenotypes, it is possible that the only time they alter gene expression is in response to an active *P. destructans* infection. Therefore, inclusion of pre‐WNS samples could skew results. In addition, only including the post‐WNS samples controls for any confounding factors related to analyzing expression across these two time periods. We called SNPs and filtered RNA reads as described above and performed the eQTL analysis in MatrixEQTL with the same parameters as discussed previously.

In all cases, we considered a SNP‐gene relationship significant if the false discovery rate (FDR) value was lower than 0.05 by convention. After significant SNP‐gene relationships were produced, we annotated and connected each gene to a function using the GeneCards database (Rebhan et al., 1998). GeneCards is an online database that automatically compiles information about each gene from several other databases, including SWISS‐PROT, OMIM, Genatlas, and GDB (Rebhan et al., 1998). We scanned through these annotations to find genes that were associated with functions that might be relevant to WNS pathophysiology. We maintained a broad definition of WNS relevance both because regulatory mechanisms can be difficult to predict and because the molecular pathways underlying WNS disease progression are not fully elucidated. We considered any gene associated with immune function, metabolism, circadian rhythms, torpor, or hydration to have potential relevance to disease outcomes, but we recognize that proteins can have varying functions and that genes outside of these associations could be meaningful to survival from WNS.

### 2.6 Target and random region SNP eQTL

#### 2.6.1 Variant calling

We used *angsd* to call SNPs within all sequenced regions, including the target SNPs themselves, regions flanking the target SNPs, and regions randomly pulled from the genome. It is typical for eQTL analyses to limit tests to targeted SNPs to reduce noise and increase statistical power, especially when sample size is relatively small (Grabek et al., 2019), but we included this larger analysis to determine if the target SNPs still presented significant relationships when there were many tests performed and if any loci in close proximity had stronger relationships than their respective target SNP. We used the same exclusion principles as the target SNP call except the minimum MAF was 20%, the minimum number of individuals was 100, the minimum minor allele homozygote count was 10, and SNPs were required to be in Hardy–Weinberg proportions. We also used these SNPs to analyze population structure, as described in further detail below. Finally, we used ngsLD and R to calculate linkage disequilibrium and construct a linkage disequilibrium decay plot for the SNPs within the target and random regions (Fox et al., 2019).

#### 2.6.2 eQTL analysis

We performed a second eQTL analysis on the full complement of SNPs. In this analysis, we simultaneously scanned target SNPs, the 1000‐bp regions flanking the target SNPs, and regions randomly pulled from the genome for eQTLs. The goal in scanning the target regions was to identify SNPs with WNS‐relevant functions that could have been under selection but were masked in the Gignoux‐Wolfsohn et al. (2021) analysis by being in LD with any of the target SNPs. We once again used MatrixEQTL with an ANOVA model to perform this analysis with the same parameters as described for the target SNP eQTL. We used sex, hibernaculum, season, period, and RNA sequencing batch as covariates.

### 2.7 Population genetics

The population substructure among samples has the potential to impact the analysis. Past work suggests *M. lucifugus* populations are panmictic in evolutionary time across much of North America east of the Rocky Mountains (Dixon, 2011; Gignoux‐Wolfsohn et al., 2021; Vonhof et al., 2015; Wilder et al., 2015). However, several analyses have found evidence of population substructure among fall swarming sites, although it does not always relate to geographic distribution and gene flow is high across most of the range (Dixon, 2011; Johnson et al., 2015; Miller‐Butterworth et al., 2014; Vonhof et al., 2015; Wilder et al., 2015). We included sampling site as a covariate in each eQTL analysis to control for the effects of population subdivision. We also evaluated the potential population structure in the current study by employing a PCA on the full suite of SNPs from the target region analysis using the program PCAngsd (Meisner et al., 2021). In addition, we performed an admixture analysis using the program NGSadmix (Skotte et al., 2013), assigning *K* values (number of populations) of 1–8 with 5 replicates each. We then compared the log‐likelihood and Dk, the average proportion of an individual's genotype belonging to 1 population, between models with different values of *K*. The value of *K* that minimized log‐likelihood and maximized Dk would be considered the most strongly supported value of *K*.

## 3 RESULTS

### 3.1 Target SNP eQTL

#### 3.1.1 Variant calling and gene expression

Genotyping resulted in 46 of the 63 target SNPs in our probes with sufficient quality and between‐sample variability to be included in the analysis. A total of 37,518,634 reads remained in the expression dataset after final filtering, and samples had 255,229 ± 8269 (mean ± SEM) reads. In total, 7969 genes were expressed at some level after all the filtering steps. The PCA of the expression count data indicated no obvious outliers, although there was strong separation along principal component 1, which explained 69.4% of the variation in the expression data (Figure S1).

#### 3.1.2 Target SNP eQTL analysis

After filtering, 129 samples were retained with sufficient SNP and expression data for the isolated target SNP eQTL analysis. Of the 9 cis‐ and 4368 trans‐eQTLs that were under the p‐value thresholds of 2 × 10^−2^ and 1 × 10^−2^, respectively, 0 cis‐ and 46 trans‐eQTLs returned an FDR <0.05 (Table 1), including 20 of the target SNP loci. Approximately 85% of the eQTLs were associated with expression at annotated genes with functions including innate immunity, homeostasis, metabolism of various macromolecules, antigen recognition, and the regulation of transcription (Table 1). Most genes (33 of 40) decreased in expression with the allele associated with WNS selection (Table 1). Some of the target loci had low numbers of minor allele homozygotes and had significant relationships (Table S4). We chose to include the results of these comparisons, but denote any with less than 5 minor allele homozygotes with an asterisk in Table 1 and list all minor allele homozygote counts in Table S4.

**TABLE 1:** A truncated list of the significant eQTL–gene relationships under an FDR of 0.05 from the isolated target SNP eQTL.

SNPID	Loc	Analyses	FDR	Gene	Regulation	Abv. Function
G55	trans	Both	0.000142	POLR3K	Down	Innate immune response, interferon I and NF‐kβ
G49	trans	Both	0.000514	UBE2E3	Down	Class I MHC
G50	trans	Both	0.012	ENPP4	Down	Innate immune system, blood coagulation
G17	trans	All sample	0.027	SEC61A1	Down	Class I MHC, antigen cross‐processing
*G47	trans	Both	0.031	SLA	Down	Innate immune response, t‐cell receptor, cytokine signaling
G44	trans	All sample	0.038	CD2BP2	Down	Antigen recognition, t‐cell activation
G51	trans	All sample	0.038	EIF2AK2	Down	Immune response to viruses, interferon, NF‐kβ, cytokines, insulin signaling
G02	trans	Post	0.023	GNG11	Up	Anti‐inflammatory cytokine production
*G55	trans	Post	0.030	SOCS1	Down	Cytokines, type I and type II interferon, class I MHC
G42	trans	Post	0.032	ADGRE3	Down	Innate immune system, inflammatory response
G05	trans	Post	0.032	OTUD5	Up	Type I interferon
G14	trans	Both	0.0069	CDC42	Up	Phagocytic cup formation
G51	trans	All sample	0.026	GRAMD1C	Down	Cholesterol metabolism, responds to lipid levels
G51	trans	All sample	0.026	EIF6	Up	Insulin/glucagon
G15	trans	All sample	0.038	ABHD4	Up	Metabolic process, lipid homeostasis
G06	trans	Post	0.032	MRPS31	Down	Type I diabetes
G14	trans	Both	0.026	TIGAR	Up	Metabolic and oxidative stress
G13	trans	All sample	0.045	AK4	Down	Oxidative stress
*G51	trans	Post	0.00021	PER3	Down	Core component of circadian rhythm, metabolism, body temperature, endocrine, immune, and cardiovascular systems
G06	trans	Post	0.023	GFPT1	Down	Regulates the expression of circadian clock genes and metabolic fluctuations

*Note*: A full list is available in Table S4. SNPID refers to the associated target SNP number from Table S2. An asterisk in front of the SNP ID denotes that the associated SNP has five or fewer minor allele homozygotes. Loc indicates whether the gene was located in cis or trans to the SNP. Analyses refer to which analysis (all samples or post only) the relationship was derived from. FDR is the statistic for each relationship, and the posted FDR for relationships that came up in both analyses is from the all‐samples analysis. “Gene” is the name of the associated gene. Regulation describes whether the gene shows increased (UP) or decreased (DOWN) expression with the putatively positively selected allele of the relevant SNP in WNS‐infected populations. The functions are truncated summaries from the genecard annotations for each gene.

#### 3.1.3 Post‐WNS target SNP eQTL analysis

Within post‐WNS samples, 45 SNPs maintained adequate quality and between‐sample variability to be included in the analysis. The eQTL analysis included 93 of the 99 total post‐WNS samples and yielded 0 cis‐ and 77 trans‐eQTLs under an FDR of 0.05 (Tables 1 and S4). These relationships were related to 21 of the target loci, with trans‐eQTLs related to the expression of genes associated with responses to DNA damage, circadian rhythms, the innate immune system, cell signaling, and the cell cycle (Tables 1 and S4). There were 19 post‐WNS eQTLs that overlapped with the pre‐ and post‐WNS eQTLs, and 16 of the target loci were represented in both analyses. In total, between both isolated SNP analyses, we found 105 relationships, including 25 target SNPs and 102 unique genes.

### 3.2 Target and random region eQTL

#### 3.2.1 eQTL analysis

**FIGURE 2:**
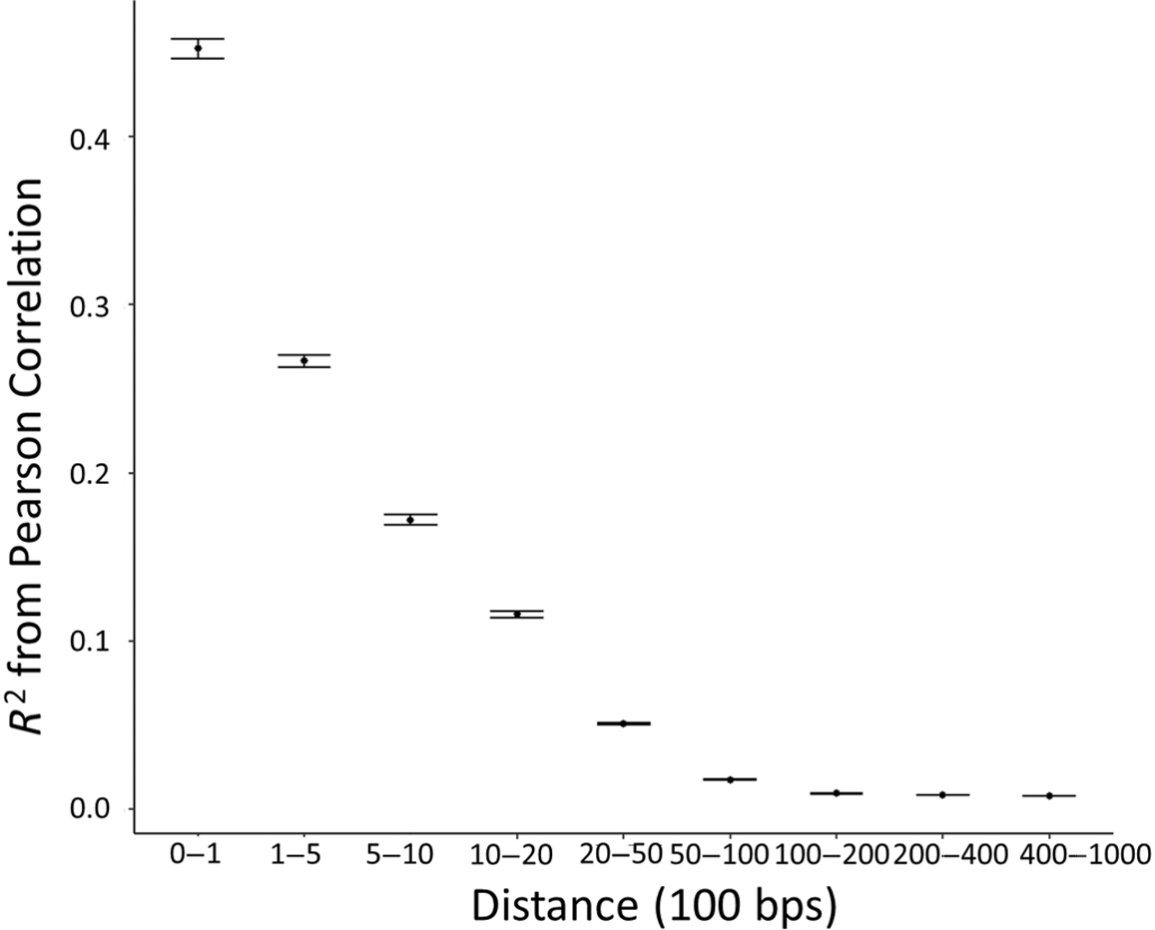
Linkage disequilibrium decay plot depicting the correlation between genomic distance (where 1 = 100 base pairs) and the *R* ^2^ value of the Pearson correlation between SNPs. *R* ^2^ values are averaged over continuously increasing bins of distances. The point represents the mean *R* ^2^ for each bin, and the error bars are the 95% confidence intervals.

We found 10,209 SNPs that passed all filtering steps across the target and random regions contained in our dataset; these included 30 target SNPs and 519 loci that occurred within target regions. As expected, we observed exponential decay of linkage disequilibrium with distance between SNPs, dropping below an average *R* ^2^ of 0.1 after the 1000–2000 bp bin (Figure 2). The full complement eQTL analysis contained 134 samples with sufficient SNP and expression data. We uncovered four relationships under an FDR of 0.05 to three target SNPs and 74 relationships to 51 loci in the target regions. The target SNPs G05, G06, and G42 all showed relationships to the same genes as in the isolated target SNP analysis. Two of these relationships were also significant in the all‐sample isolated target SNP analysis and the post‐WNS‐only isolated target SNP analysis. Genes related to loci in the target regions had multiple functional annotations, including the innate immune system, cell signaling, pathogen recognition, DNA repair, metabolism, transcriptional regulation, and the cell cycle (Tables 2 and S5). In comparison, random regions contained 1566 significant eQTL relationships relating to 1163 of the 9660 loci. No loci, whether target SNPs, SNPs in target regions, or SNPs in random regions, had any cis relationships under an FDR of 0.05.

**TABLE 2:** Significant eQTL–gene relationships under an FDR of 0.05 from the target region eQTL.

Scaffold	Position	SNPID	Near Target SNP	Distance (bps)	FDR	Gene	Function
GL429768	9,041,952	G06	NA	0	0.015	DIAPH1	Hearing loss, cell cycle
GL429768	29,962,249	G05	NA	0	0.018	PGAP6	Phospholipase activity
GL429955	1,484,586	G42	NA	0	0.046	MED4	Transcription
GL430121	936,613	T00635	G51	29	0.0032	SIGMAR1	Immune/nervous signaling, oxidative stress
GL429861	293,533	T00355	G26	161	0.0064	ILRUN	Inhibitor of inflammatory cytokines
GL430166	11,910	T00706	G54	22	0.015	CACTIN	Negative regulator of toll‐like receptors, NF‐kβ, and interferons
GL430101	497,490	T00670	G50	582	0.024	ENPP4	Innate immune system, blood coagulation
GL430008	1,395,730	T00574	G46	380	0.028	ITGB3BP	Repressor of NF‐kβ
GL429768	29,961,757	T00076	G05	492	0.034	OTUD5	Negative regulator of innate immune system, suppress type I interferon
GL429885	2,042,749	T00436	G35	781	0.038	KAT2A	NF‐kβ repressor, T‐cell activation, IL2 promotion
GL429768	29,961,970	T00083	G05	279	0.045	TLR4	Toll‐like receptor 4
GL430158	593,747	T00721	G53	94	0.050	POLR3B	RNA polymerase III subunit, induction of interferons and NF‐kβ
GL429962	688,419	T00542	G43	874	0.00933	SLC16A5	Monocarboxylate transporter
GL429885	617,655	T00427	G64	167	0.033	CAVIN2	Glucocorticoid receptor pathway, cell starvation
GL430158	593,747	T00721	G53	94	0.035	SMCR8	Regulation of macroautophagy
GL429929	2,943,907	T00498	G40	26	0.043	SCFD1	Autophagosome assembly

*Note*: This table has been truncated to only include a subset of relationships. All target region relationships were trans and are available in Table S5. Here, scaffold and position refer to the genomic locus of the SNP. The SNPID is in reference to the target region SNP that correlates with the gene in that row. Near Target SNP is the ID of the closest target SNP (as shown in Table S3), and distance is the number of base pairs between the target region SNP and the target SNP. FDR is the statistic regarding the correlation between the SNP and the gene. The functions are summaries of the genecard annotations for each gene.

### 3.3 Population structure

**FIGURE 3:**
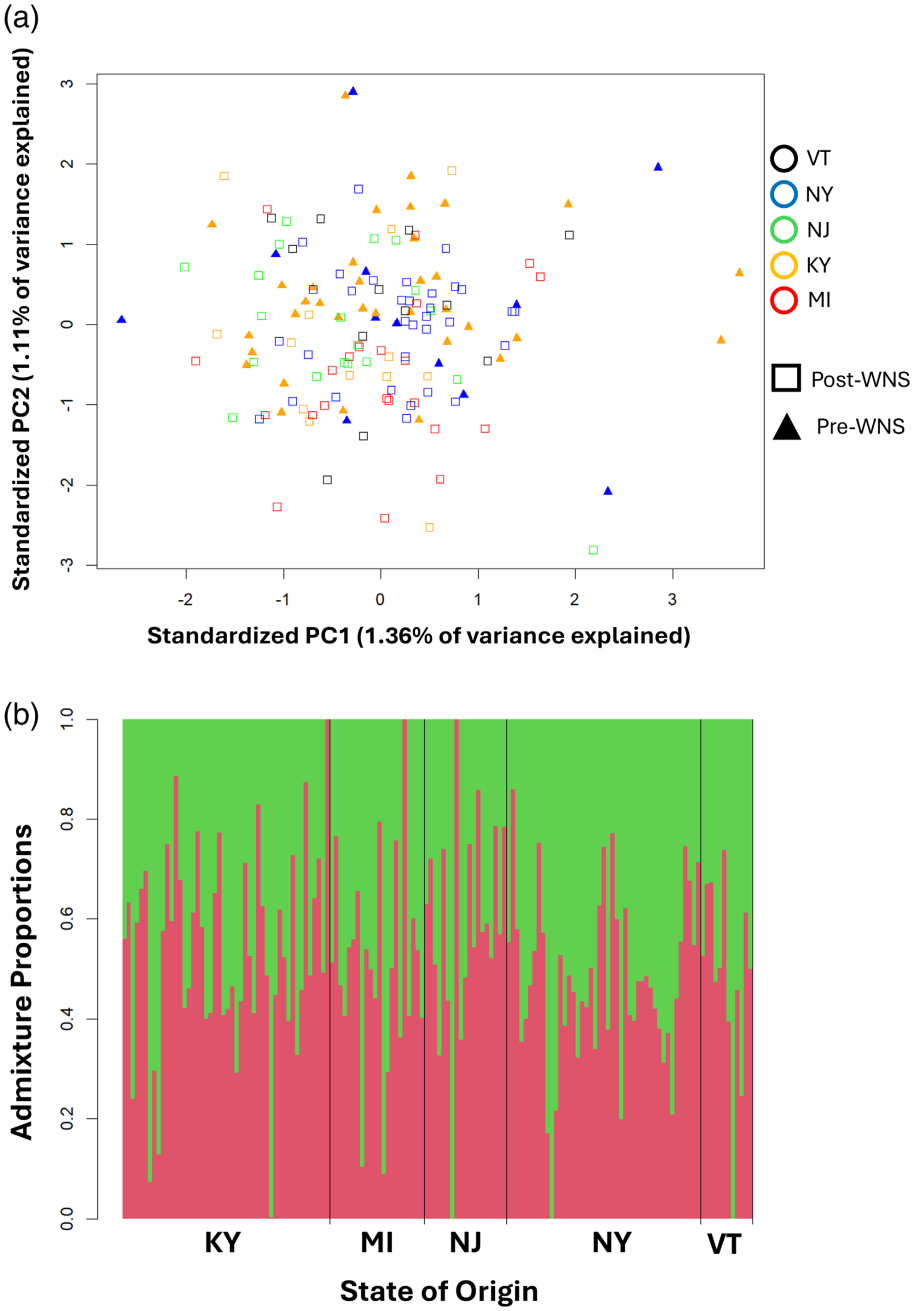
(a) A biplot showing the standardized and centered 1st and 2nd principal components of the SNP data PCA. The color delineates the state of origin of the sample. Shapes indicate whether the sample was collected before or after the arrival of white‐nose syndrome. (b) Admixture plot when the number of populations is set to 2. Each unit on the x‐axis is an individual, and the y‐axis is the proportion of each individual's SNP profile that aligns with each theoretical population.

The PCA explained a very small amount of variation, and the top principal components did not correlate with geographic distance, suggesting low amounts of population substructure based on hibernaculum or region (Figure 3a). The comparison of admixture models supported a *K* value of 1, with that model possessing the lowest log‐likelihood. The Dk value most strongly supported a *K* value of 2, although that analysis is unable to consider a *K* value of 1. The *K* value optimization and admixture plots demonstrate low levels of population structure across the sampled *M. lucifugus* populations (Figure 3b).

## 4 DISCUSSION

### 4.1 Target SNP eQTLs

We completed two distinct analyses examining loci putatively under WNS‐induced selection in *M. lucifugus* to both validate their status as targets of selection and provide evidence to support their phenotypic function. We found significant changes in gene expression within wing tissue related to 25 of the included 46 (~54%) SNPs identified in a previous GWSS. These loci were significantly involved in 105 relationships, correlating with the expression of 102 unique genes. Annotations suggest that their function is relevant to WNS pathophysiology, including 20 genes related to the immune system, two associated with circadian rhythms, six with cellular responses to stress, and 11 related to nutrient cycling, metabolism, and starvation.

Interestingly, ~75% of genes with significant relationships to target SNPs showed a decrease in expression, with the allele putatively experiencing positive selection. This pattern is consistent with gene expression studies comparing species of differential disease susceptibility, with more resistant species exhibiting a muted transcriptional response relative to susceptible ones (Eskew et al., 2021). For example, susceptible wood frogs (*Rana sylvatica*, Le Conte, 1825) demonstrated a significant rise in epidermal expression of adaptive immune genes and metabolic function upon infection with the fungal pathogen *Batrachochytrium dendrobatidis* (Berger, 1998), while resistant American bullfrogs (*Lithobates catesbeianus*, Frost, 2011) exhibited no notable changes (Eskew et al., 2018). This phenomenon has also been observed in bat species infected with WNS, with significant increases in overall gene expression and upregulation of immune‐related genes in naive *M. lucifugus* and less significant change in European *M. myotis* (Borkhausen, 1797) (Davy et al., 2020; Lilley et al., 2019; Moore et al., 2013), a resistant species that likely co‐evolved with *P. destructans* (Martínková et al., 2018; Zukal et al., 2016). Similarly, the more resistant Nearctic species *Eptesicus fuscus* (Beauvois, 1796) employs a robust local response but a muted systemic response compared to *M. lucifugus* (Davy et al., 2020; Frank et al., 2014). Our findings suggest that this muted expression pattern also occurs within species, where overall gene expression is decreased in genotypes with lower susceptibility.

The immune system has evolved to protect organismal function (Du Pasquier, 1993; Travis, 2009); therefore, a reduced immune response may seem counterintuitive in promoting survival during disease. However, several competing hypotheses offer compelling reasons for such a phenomenon to occur. A successful initial immune response may eliminate or greatly reduce pathogen loads, removing the need for continued upregulation of immune genes (Eskew et al., 2021). Alternatively, resistance may be generated by means other than an immune response. In the case of an epidermal disease, pathogen spread could be curtailed by a less penetrable integument or by the competitive activity of the resident microbiome (Eskew et al., 2021). Indeed, growth of *P. destructans* is limited on the wings of *M. myotis* despite a lack of aggressive immune response (Davy et al., 2017, 2020; Lilley et al., 2019) and microbes that impact *P. destructans* growth have been isolated from *Eptesicus fuscus* (Cheng et al., 2017; Hoyt et al., 2015).

The examples suggested above are methods of host resistance, where the growth or virulence of the pathogen is hampered to promote the survival of the host. However, host tolerance can also produce muted gene expression responses in infected animals (Eskew et al., 2021). Under a host tolerance scenario, survival is improved by reducing the negative effects of infection despite continued pathogen growth. In the case of WNS, changes in immune expression, higher pre‐hibernation fat mass, and longer torpor bout intervals in *M. lucifugus* despite unchanged pathogen loads are mechanisms of host tolerance that likely act to promote survival (Cheng et al., 2019; Lilley et al., 2016, 2019). Our findings suggest some support for mechanisms of host tolerance in *M. lucifugus* through loci relationships to the expression of genes associated with circadian rhythms, metabolism, and endocrine signaling in energy systems. The reduced immune activity we observed could itself be a form of host tolerance. In infected bats, disease typically emerges in wing tissue, triggering a systemic response of torpor pattern disequilibrium and increased energy expenditure (Cryan et al., 2010; Verant et al., 2014; Warnecke et al., 2013). If that response is altered such that the pathological metabolic increase does not occur or is reduced, immune repression could act to increase host tolerance without influencing fungal growth. Indeed, this mechanism of tolerance is likely active in palearctic bats that demonstrate reduced immune responses without influence on pathogen growth (Davy et al., 2020; Lilley et al., 2019; Whiting‐Fawcett et al., 2024), although there are some European species that appear to incur resistance via a robust, successful immune response (Li et al., 2023; Whiting‐Fawcett et al., 2024). Regardless, tolerance and resistance likely exist across a spectrum in most cases, and a species may demonstrate altered responses over time (Fornoni et al., 2004; Restif & Koella, 2004; Whiting‐Fawcett et al., 2024). Previous transcriptomic studies have found little evidence of resistance or tolerance in surviving *M. lucifugus* populations (Cheng et al., 2024; Lilley et al., 2019). However, high variation in individual responses could obscure results (Eskew et al., 2021), and phenotypic studies suggest increased tolerance in at least some populations (Cheng et al., 2019, 2024; Lilley et al., 2016, 2019). A transcriptomic comparison between *M. lucifugus* from surviving populations and naïve ones showed some differences, but whether expression increased or decreased varied by gene and did not mimic that of palearctic species (Lilley et al., 2019). Our results are consistent with the findings of that comparison, which suggested potential selection for an altered immune response in surviving populations. Overall, it is likely that *M. lucifugus* employs a mixed and context‐dependent strategy (Cheng et al., 2024; Langwig et al., 2012; Whiting‐Fawcett et al., 2024), but further study is needed to fully understand the response of this species to WNS.

Functional annotations of the related genes can also provide insight into the regulatory impact of the SNP alleles putatively experiencing positive selection. Multiple immune genes for which we observed differential expression related to target SNPs had direct links to the previously established transcriptomic response of *M. lucifugus* to WNS. Several had general links to pathogen/antigen recognition and processing, and early immune defense. Also impacted were pathways associated with NF‐kβ, a transcription factor often induced following pathogen recognition, which activates transcription of cytokines and interferons (a subclass of cytokines that attack invading pathogens). Released cytokines typically then activate other aspects of immune responses, as well as the inflammasome (Field et al., 2018; Lilley et al., 2019). In our study, multiple genes showed differential expression associated with the target SNPs that related both to the activity of cytokines and interferons, as well as the process of inflammation, suggesting that these loci impact the immune system either through direct changes in the responsiveness of cells to pathogen invasion or through a downstream result of some other process.

Surprisingly, some of the associated immune genes, particularly those related to responses to intracellular bacterial and viral pathogens, major histocompatibility complex class I (MHC class I) antigen presentation and the interferon type I response, are not typically associated with responses to extracellular pathogens. MHC class I molecules are located on the surface of all nucleated cells and react to pathogens sourced from inside the cell (i.e., viruses and intracellular bacteria, Hewitt, 2003; Neefjes et al., 2011; Rock et al., 2016). Exterior cellular pathogens (i.e., fungal dermatitis) are usually addressed by MHC class II molecules (Neefjes et al., 2011; Rock et al., 2016). However, cross‐presentation, where antigens derived from outside the cell are presented by MHC class I molecules, does occur, although most studies have focused on this phenomenon in “professional” antigen‐expressing cells of the immune system and in relation to viruses (Kurts et al., 2010; Rock et al., 2016). Although, evidence suggests cross‐presentation including MHC class I molecules can occur in fungal infections (Speakman et al., 2020), and one of the genes (SEC61A1) we found to be differentially expressed is associated with antigen cross‐presentation. Similarly, type I interferons are most often associated with viral infections, although they also play a role in fungal infections (McNab et al., 2015). The type I interferon response was associated with *P. destructans* infection in a previous transcriptomic study, offering complementary validation that type I interferons are part of that pathway (Field et al., 2015). In addition to these two pathways, several differentially expressed genes were associated with viral budding (MVB12A) or the sensing of intracellular bacteria and viruses (POLR3K, EIF2AK2; Rebhan et al., 1998).

Differential expression related to the target SNPs of several genes involved in responses to intracellular bacteria and viruses presents possible evidence that these loci impact survival through the *M. lucifugus* response to secondary infection. As *P. destructans* invades the tissue of hibernating bats, it disrupts the skin barrier and allows entry of other pathogens that would not normally have access (Verant, 2016). Supporting this hypothesis, several bacterial and fungal genera were found to be widespread on bats with active WNS while being nearly absent from uninfected individuals (Verant, 2016). Most of these genera are rarely pathogenic organisms outside of opportunistic invasions (Verant, 2016). In addition, *P. destructans* infection results in increased replication of at least one coronavirus that naturally occurs in bat hosts (Davy et al., 2018). The impact of secondary infection on WNS pathophysiology is not clearly understood, but our findings of SNPs associated both with survival and responses to intracellular pathogens may suggest secondary infection has an important impact on disease outcome. However, given previous relationships between some of these pathways and fungal infection, it is possible transcriptional differences are directly associated with the response to *P. destructans* (McNab et al., 2015; Speakman et al., 2020). In either case, this system may represent an interesting case study of the relationships between MHC class I, interferon I pathways and fungal infection in a non‐model organism.

Expression of genes that relate to metabolic functions, such as insulin signaling, lipid homeostasis, and starvation, was also impacted by the target SNPs. Given that premature depletion of energy is a leading cause of mortality in WNS‐affected bats and that transcriptomic evidence supports a metabolic response to *P. destructans* infection in wing tissue (Cheng et al., 2019, 2021; Field et al., 2018; Hoyt et al., 2021; Lilley et al., 2019; Whiting‐Fawcett et al., 2021), it is possible that changes in their expression either alter energy cycling or are a sign of better body condition during late hibernation. Some of the target SNPs themselves have been implicated in the metabolic system (Gignoux‐Wolfsohn et al., 2021), and other studies of WNS‐induced selection in *M. lucifugus* have uncovered loci in regions related to these processes (Auteri & Knowles, 2020; Lilley et al., 2020).

Lastly, we found eQTLs related to reduced expression of two genes (GFPT1 and PER3) responsible for regulation of circadian rhythms. PER3 is thought to be a core component of circadian rhythm regulation and a regulator of other relevant functions such as metabolic, immune, and endocrine systems (Pendergast et al., 2012; Rebhan et al., 1998; Yeom et al., 2018). The PER genes are active in the suprachiasmatic nuclei (circadian control center of the brain), but PER3 is also active in tissues related to the peripheral circadian system, including skin keratinocytes (Pendergast et al., 2012; Yeom et al., 2018). Patterns of torpor arousal are hypothesized to be controlled, in part, by circadian rhythms in rodent hibernators (Malan, 2010), although not based on the body temperature or light–dark cycles indicative of typical suprachiasmatic nucleus control of clock genes (Ikeno et al., 2017; Malan, 2010). Less is known of the circadian influence on torpor patterns in bat hibernators, but clock genes have been hypothesized to have an impact (Willis & Wilcox, 2014). Given the pathological increase in torpor arousal frequency indicative of WNS, changes in circadian control of torpor arousal could certainly impact the survivorship of infected individuals.

That 46% of target SNPs did not demonstrate significant associations is not overly surprising. It is common for studies that connect loci putatively under selection to phenotypic traits to only find relationships for some target SNPs. For example, 8 out of 14 loci (57%) associated with the onset of hibernation in 13‐lined ground squirrels (*Ictidomys tridecemlineatus*, Mitchill, 1821) were found to relate to changes in gene expression among heart, liver, skeletal muscle, and brown adipose tissues (Grabek et al., 2019). Similarly, two out of 17 loci (12%) identified as putatively associated with mountain pine beetle (*Dendroctonus ponderosae*, Hopkins) susceptibility in lodgepole (*Pinus contorta*, Louden) and jack pines (*Pinus banksiana*, Banks) within Canada were associated with significant changes in transcript abundances in attacked trees (Cullingham et al., 2020). Our study considered only wing tissue; gene expression is both tissue‐ and condition‐specific (Maniatis et al., 1987; Sonawane et al., 2017; Whitehead & Crawford, 2005). At this juncture, we cannot determine whether the SNPs without relationships were false positives within the GWSS or perhaps active in other tissues. Further studies attempting to link these loci to disease‐relevant phenotypes are warranted.

### 4.2 eQTLs in regions surrounding target loci

We found significant differential expression related to 51 loci occurring within 1000 bp of 34 target SNPs. Due to proximity, it is likely that these loci exist in a state of LD with nearby target SNPs. Supporting this linkage, we found that average *R* ^2^ correlations between SNPs tended to remain above 0.1 until they were ~2000 bp apart. Interestingly, 26 of these loci showed significant relationships to gene expression, while the target SNPs with which they are in LD showed none despite being present in the analysis. For example, one locus near target SNP G05 replaced the target SNP's relationship to gene OTUD5 from the isolated target SNP analysis. Such examples of nearby loci having stronger relationships to gene expression than the target loci may suggest that at least some target SNPs were masking true loci under selection. Although such a claim requires further validation, it does suggest these nearby loci are worthy of further attention. It is also true that a function related to SNPs in close proximity does not necessarily mean the nearby target SNP has no function. In fact, if the target SNPs are indeed components of regulatory units, the surrounding loci are likely to be part of the units as well because the lengths of regulatory loci can be over 1000 base pairs (Blackwood & Kadonaga, 1998; Li & Wunderlich, 2017; Yáñez‐Cuna et al., 2013). Allelic variation of any base pairs within regulatory units could have complex impacts on function.

Additionally, these genes had functions associated with WNS pathophysiology, with 19 relating to the immune system, 11 to metabolism, and six to cellular responses to DNA damage and stimuli. Some significantly associated genes have similar functions to those showing altered expression in the isolated target SNP analysis. For example, there were again multiple genes related to the transcription factor NF‐kβ and cytokine production. We also uncovered new functions related to the innate immune system, including two genes associated with toll‐like receptors, one being a receptor itself (TLR4) and the other a negative regulator of toll‐like receptors (CACTIN) (Rebhan et al., 1998). Toll‐like receptors are one of the primary receptors known to interact with fungal pathogens in mammals, including *P. destructans* (Davy et al., 2020; Field et al., 2015, 2018; Romani, 2011; Whiting‐Fawcett et al., 2021). Metabolism related genes also demonstrated similar patterns to the isolated target SNP analysis through association with macroautophagy and lipid homeostasis.

Interestingly, the eQTL including SNPs from all regions yielded fewer significant relationships to target SNPs than the isolated target SNP analysis. This, in part, could be due to fewer target SNPs being included in the analysis, with only 30 loci in the full analysis versus 46 in the target SNP eQTL. Out of necessity to maximize statistical power for our sample size, we used slightly more stringent SNP filtering criteria in the full complement eQTL analysis, which may have contributed to differences in significance among analyses. In addition, there were comparatively many more alternative SNPs in the other regions (519 target regions, 9660 random regions), which can overshadow the impact of target loci, especially if their effect is subtle or context dependent. The large number of alternative SNPs also led to two orders of magnitude more tests (~80,000,000 vs. ~400,000), likely underpowering some relationships. For these reasons, it is not unusual for eQTL studies to limit analysis to loci of interest, as we did for the isolated target SNP analysis (Grabek et al., 2019). However, we chose to include the full complement of SNPs in this part of the analysis to compare target loci to the loci with which they are in LD.

Random regions showed more significant relationships than target regions (1566 vs. 74, respectively), although at a similar proportion to the number of SNPs from each included in the analysis (9660 vs. 524). The larger number of loci in random regions provided more opportunity for relationships and consequently a higher chance of false positives despite efforts to control them via adjusted *p*‐values (FDR). However, it is equally likely that many or most of these relationships are legitimate and the involved loci are part of coding or regulatory sequences. We did not avoid coding regions when constructing our random probes; it is possible that some of these SNPs impact protein structure. In addition, it is increasingly recognized that regulatory loci are relatively common among genomic sequences. For example, over 70,000 regulatory loci have been discovered in the human genome to date (Buniello et al., 2019; Claussnitzer et al., 2020; Degtyareva et al., 2021), and 147,419 SNPs were revealed as eQTLs in the buds of a population of the forest tree *Populus tremula* (Linnaeus, 1753) in Sweden (Mähler et al., 2017). Given that we scanned ~10,000 loci in the random regions, it is not surprising that we uncovered some that result in transcriptional variation.

### 4.3 Limitations

eQTL analyses ideally consist of several hundred or even thousands of samples to maximize statistical power (Shi, 2020). In free‐living non‐model organisms, difficulty in obtaining samples coupled with a lack of control over environmental variability results in limitations. The study also would have benefited from an analysis of infection status in the post‐WNS samples, as infection status leads to large differences in gene expression (Davy et al., 2020; Field et al., 2015, 2018; Lilley et al., 2019) that could obscure results. However, previous studies imply the vast majority of bats in affected hibernacula are infected, suggesting it is likely the post‐WNS samples used in this study were from infected individuals (Hoyt et al., 2020).

Finally, the geographic range of our samples presents the opportunity for population structure to influence results. However, our analyses suggest very little population substructure relating to geography among our samples (Figure 3). These results largely agree with past work suggesting high levels of gene flow among *M. lucifugus* in the Eastern and Midwestern United States (Gignoux‐Wolfsohn et al., 2021; Miller‐Butterworth et al., 2014; Vonhof et al., 2015; Wilder et al., 2015). Although past work also suggests substructure among hibernacula and fall swarming sites as well as substructure separating the eastern and western USA, this structure did not appear directly related to geographic distribution in the east (Johnson et al., 2015; Miller‐Butterworth et al., 2014; Vonhof et al., 2015; Wilder et al., 2015). Likewise, we did not see strong genetic structure relating to geographic location in our samples.

### 4.4 Future research directions

Here we show the first evidence linking loci from a GWSS of WNS‐impacted bats to phenotypic function. Future analyses would benefit from including the loci or genomic regions discovered in other published studies. The fact that few loci are shared between the results of these analyses may provide insight into potential polygenic evolution across the range of *M. lucifugus*. It would also be prudent to evaluate the impact on the transcription patterns of other tissues to reveal how these SNPs behave in other contexts. Future studies could also search for impacts of SNPs at other physiological scales, such as protein expression or metabolic function. Given that mechanisms of response can change over time (Cheng et al., 2024) and selective pressure from WNS is dependent on environmental context (Hoyt et al., 2021; Langwig et al., 2012), determining how time and environment impact adaptation would be valuable.

## 5 CONCLUSIONS

Population recovery, phenotypic changes, and genomic loci previously identified as putatively under selection suggest rapid adaptation to WNS in some populations of *M. lucifugus*. Here, we provide the first evidence linking some of these potentially selected loci to phenotypic mechanisms associated with disease survival. We found changes in the expression of 102 genes associated with 54% of the SNP loci discovered in the analysis by Gignoux‐Wolfsohn et al. (2021). Some of these genes had functions related to WNS survival through association with the immune system, circadian rhythms, metabolism, cellular stress, or energy cycling. Nearly 75% of genes decreased in expression with the putatively beneficial allele of their associated locus, reminiscent of patterns found in species less susceptible to disease. We also found significant eQTLs near the target SNPs, suggesting that these adjacent loci are under selection but were masked from previous analysis due to linkage disequilibrium or are part of the same regulatory units as the target SNPs. We see our work as the first step in uncovering the mechanistic connection between these putatively beneficial loci and WNS survival in *M. lucifugus*.

In addition, our findings provide validating evidence of the beneficial functions of the putative targets of selection during the spread of the WNS infection. Genome‐wide scans for selection (GWSS) are critical studies for discovering loci under selection but are susceptible to false positives. Studies at multiple physiological scales can support or oppose the findings of a GWSS in a framework known as complementary validation. In finding relationships to gene expression in some of these loci, we provide further support for previous GWSS findings. Confidence in the benefit of SNP alleles to survival is critical to management efforts aimed at determining population genetic susceptibility and the potential transplant of more resistant individuals.

## CONFLICT OF INTEREST STATEMENT

The authors of this manuscript have no conflicts of interest to report.

## FUNDING INFORMATION

The Eppley Foundation for Research, United States Fish and Wildlife Service (grant: #F15AP00949), Rutgers Institute of Earth, Ocean and Atmospheric Sciences, Rutgers Department of Ecology, Evolution and Natural Resources.

## Supporting information


Appendix S1.

## Data Availability

Sequences are available from the NCBI Short Read Archive (BioProjectID PRJNA1115790). All scripts and notebooks are publicly available in a GitHub Repository BKrek89/MYLU_eQTL (github.com).
