# Supplementary material for: Impact of putatively beneficial genomic loci on gene expression in little brown bats (Myotis lucifugus, Le Conte, 1831) affected by white‐nose syndrome
Source: Evol Appl. 2024 Sep 19;17(9):e13748. doi: 10.1111/eva.13748 (PMC11413065; doi:10.1111/eva.13748)
Supplement: Supplementary file 1 — Appendix S1. [file EVA-17-e13748-s001.zip › Captions_S4S5.docx]

**Supplementary Table 4.**

A list of all eQTL-gene relationships under an FDR of 0.05 from the isolated target SNP analysis. The analysis column denotes whether the relationship was discovered in the all sample analysis which included both pre- and post-WNS samples, or the post analysis which only included post-WNS samples. The SNP column refers to the associated target SNP number from suppl. table 2. MA homozygote shows the number of minor allele homozygote samples for that locus. The reg column refers to whether the gene was located in cis or trans to the SNP. The gene column shows the annotated gene ID from the Myoluc_2.0 genome for each related gene. The up or down with selective allele column shows the direction of regulation of each gene. F-test, p-value, and FDR are statistical outputs from Matrix-eQTL for each relationship. The columns genename and genecards function show the common name for each gene ID and a truncated description of each gene’s function from genecards database descriptions, respectively.

**Supplementary Table 5.**

A list of all eQTL-gene relationships under an FDR of 0.05 within target regions from the target region eQTL analysis. The SNP column refers to the ID designated to each locus. Those starting with a G are target SNPs from suppl. table 2 and those starting with a T are arbitrarily numbered loci from the target regions. Scaffold and position define the genomic coordinates of each locus. The gene column designates the annotated gene ID from the Myoluc_2.0 genome. F-test, p-value, and FDR represent the statistical output for each relationship from Matrix-eQTL. Closest target SNP and distance to target SNP report the closest target SNP to the eQTL locus reported as the SNP ID from suppl. table 2 and the distance in base pairs between the target region and target loci. The columns genename and genecards function show the common name for each gene ID and a truncated description of each gene’s function from genecards database descriptions, respectively.
