# Supplementary material for: Impact of putatively beneficial genomic loci on gene expression in little brown bats (Myotis lucifugus, Le Conte, 1831) affected by white‐nose syndrome
Source: Evol Appl. 2024 Sep 19;17(9):e13748. doi: 10.1111/eva.13748 (PMC11413065; doi:10.1111/eva.13748)
Supplement: Supplementary file 1 — Appendix S1. [file EVA-17-e13748-s001.zip › eva13748-sup-0001-FigureS1.docx]

**
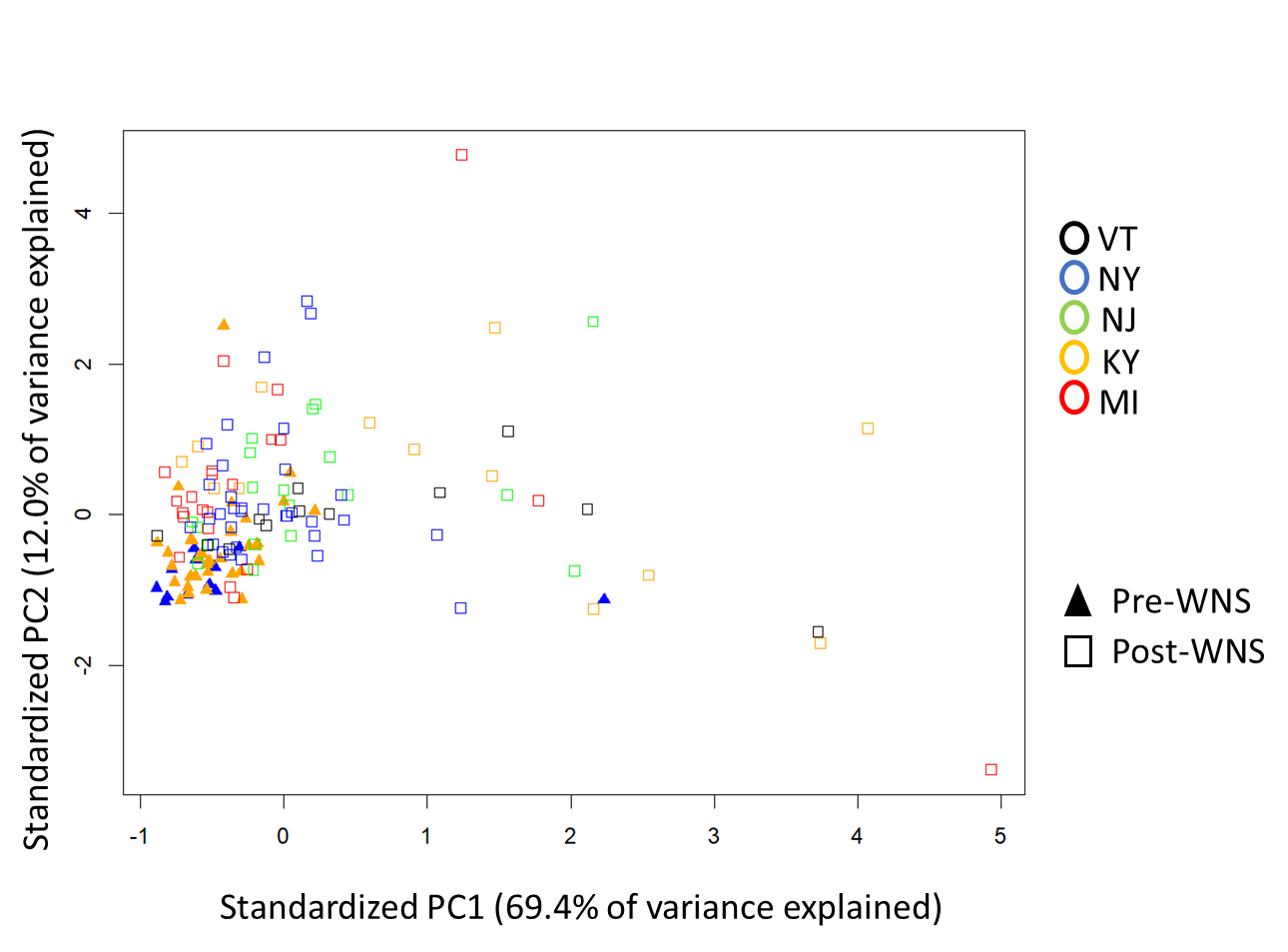
**

**Supplementary Figure 1.** Biplot of the standardized and centered first two principal components of the expression data PCA. Colors delineate the state of origin of the sample, and shapes indicate whether the sample was collected before or after the arrival of white-nose syndrome to that hibernaculum.
