# Supplementary material for: Impact of putatively beneficial genomic loci on gene expression in little brown bats (Myotis lucifugus, Le Conte, 1831) affected by white‐nose syndrome
Source: Evol Appl. 2024 Sep 19;17(9):e13748. doi: 10.1111/eva.13748 (PMC11413065; doi:10.1111/eva.13748)
Supplement: Supplementary file 1 — Appendix S1. [file EVA-17-e13748-s001.zip › eva13748-sup-0002-TablesS1-S3.docx]

|  |  | | | | | |  |
| --- | --- | --- | --- | --- | --- | --- | --- |
|  |  | | | | | |  |
| State | | Pre or Post WNS | WNS Infection Year | Sample # | Hibernaculum | Collection Date | Hibernation Period |
| Kentucky | | Post | ~2012-2015 | 13 | Colossal Cave | 03/16/2017 | Late |
| Kentucky | | Pre | ~2013 | 29 | Cave Hollow Cave | 03/26/1999 | Late |
| Kentucky | | Pre | ~2013 | 6 | Mitten Hollow Cave | 10/08/1999 | Early |
| Kentucky | | Pre | ~2013 | 1 | South Goldson Cave | 10/08/1999 | Early |
| Michigan | | Post | ~2012-2015 | 22 | Adventure Mine | 04/08/2017 | Late |
| New Jersey | | Post | ~2008-2010 | 19 | Hibernia Mine | 03/21/2016 | Late |
| New York | | Post | ~2006-2010 | 25 | Walter Williams Preserve | 04/22/2019 | Late |
| New York | | Post | ~2006-2010 | 8 | Walter Williams Preserve | 04/21/2016 | Late |
| New York | | Pre | ~2007-2009 | 12 | Barton Hill Mine | 03/22/1999 | Late |
| Vermont | | Post | ~2007-2009 | 12 | Aeolus Cave | 05/15/2017 | Late |

**Supplementary Table 1**. Hibernacula from which samples were collected and the dates of collection.

|  |  |  |  |  |
| --- | --- | --- | --- | --- |
|  | | | | |
| **SNPID** | **Scaffold** | **Position** | **Major Allele** | **Minor Allele** |
| G01 | GL429767 | 16839644 | T | A |
| G02 | GL429767 | 22649683 | T | A |
| G03 | GL429767 | 46534125 | C | T |
| G04 | GL429768 | 3921142 | G | C |
| G05 | GL429768 | 29962249 | A | G |
| G06 | GL429768 | 9041952 | A | G |
| G07 | GL429770 | 8507307 | A | C |
| G08 | GL429772 | 14993622 | T | C |
| G09 | GL429773 | 7204887 | C | T |
| G10 | GL429775 | 5556309 | A | C |
| G11 | GL429775 | 17831035 | C | T |
| G12 | GL429778 | 3561000 | G | A |
| G13 | GL429781 | 7594948 | A | T |
| G14 | GL429783 | 665407 | C | T |
| G15 | GL429792 | 753145 | C | A |
| G16 | GL429801 | 6986210 | A | T |
| G17 | GL429802 | 4091453 | C | T |
| G18 | GL429806 | 3311579 | T | C |
| G19 | GL429816 | 3229647 | G | A |
| G20 | GL429819 | 365354 | G | A |
| G21 | GL429835 | 2966433 | A | G |
| G22 | GL429841 | 3522450 | A | G |
| G23 | GL429841 | 612532 | G | A |
| G24 | GL429842 | 4124241 | A | G |
| G25 | GL429845 | 2091046 | C | T |
| G26 | GL429855 | 4401152 | G | C |
| G27 | GL429859 | 300331 | T | C |
| G28 | GL429861 | 293372 | A | G |
| G29 | GL429861 | 3065399 | A | G |
| G30 | GL429867 | 2575249 | A | G |
| G31 | GL429873 | 2056629 | T | A |
| G32 | GL429879 | 384336 | G | A |
| G33 | GL429885 | 3445677 | T | A |
| G34 | GL429885 | 617822 | C | T |
| G35 | GL429885 | 2043530 | T | C |
| G36 | GL429888 | 3508435 | A | T |
| G37 | GL429898 | 676714 | T | C |
| G38 | GL429910 | 2414078 | A | C |
| G39 | GL429927 | 1241213 | A | G |
| G40 | GL429929 | 2943933 | G | T |
| G41 | GL429930 | 2586279 | C | T |
| G42 | GL429955 | 1484586 | G | A |
| G43 | GL429962 | 689293 | A | C |
| G44 | GL429965 | 1956978 | G | A |
| G45 | GL429998 | 99324 | C | G |
| G46 | GL430008 | 1396110 | C | T |
| G47 | GL430018 | 180278 | T | A |
| G48 | GL430029 | 1661413 | A | C |
| G49 | GL430036 | 626424 | G | T |
| G50 | GL430101 | 496908 | T | C |
| G51 | GL430121 | 936584 | T | C |
| G52 | GL430156 | 916661 | C | T |
| G53 | GL430158 | 593841 | T | G |
| G54 | GL430166 | 11932 | C | T |
| G55 | GL430239 | 60325 | G | A |
| G56 | GL430239 | 60326 | T | C |
| G57 | GL430271 | 525210 | C | T |
| G58 | GL430281 | 514067 | T | C |
| G59 | GL430338 | 350547 | T | C |
| G60 | GL430482 | 173352 | C | T |
| G61 | GL430686 | 91034 | C | T |
| G62 | GL430746 | 9663 | C | T |
| G63 | GL432768 | 965 | C | T |

**Supplementary Table 2.** SNPs that were identified by Gignoux-Wolfsohn et al (2021) as putatively under selection due to white-nose syndrome and that were included in the target SNP eQTL analysis. Scaffold and position refer to the Myoluc 2.0 genome publicly available from ENSEMBL and NCBI. The red colored allele of the major and minor alleles is the one found to be positively selected in Gignoux-Wolfsohn et al (2021).

| **Period** | **Sample Size** | **Raw read count**  **(Mean ± SEM)** | **N%**  **(Mean ± SEM)** | **Mapped%**  **(Mean ± SEM)** | **Mapping Mismatch%**  **(Mean ± SEM)** |
| --- | --- | --- | --- | --- | --- |
| Pre-WNS | 47 | 4.7X10^6^ ± 4.1X10^5^ | 0.19 ± 0.005 | 78.98 ± 1.32 | 0.89 ± 0.02 |
| Post-WNS | 98 | 5.8X10^6^ ± 5.0X10^5^ | 0.21 ± 0.010 | 75.63 ± 0.80 | 0.90 ± 0.01 |

**Supplementary Table 3.** Table showing various quality control measures of the RNA expression data among Pre- and Post-WNS samples. The value N% is the percent of uncalled base pairs after sequencing. Mapped% is the percentage of reads uniquely mapped to the reference genome. Mapping mismatch% is the average percentage of bases that did not match the reference genome among aligned reads.
